# Primary Clinical Evaluation of Photodynamic Therapy With Oral Leukoplakia in Chinese Patients

**DOI:** 10.3389/fphys.2018.01911

**Published:** 2019-01-22

**Authors:** Ying Han, Si Xu, Jianqiu Jin, Xing Wang, Xiaodan Liu, Hong Hua, Xiaoyang Wang, Hongwei Liu

**Affiliations:** ^1^Department of Oral Medicine, Peking University School of Stomatology, Beijing, China; ^2^National Center of Gerontology, Beijing Hospital, Beijing, China; ^3^Beijing Anzhen Hospital, Capital Medical University, Beijing, China

**Keywords:** photodynamic therapy, leukoplakia, dysplasia, aminolevulinic acid, potential malignant diseases

## Abstract

**Background:** Photodynamic therapy (PDT) has demonstrated promising results in the treatment of oral leukoplakia. This study evaluated the clinical efficacy and side effects of PDT in the treatment of Chinese patients with oral leukoplakia.

**Methods:** Twenty-nine patients with oral leukoplakia were enrolled in this study, including patients with both homogenous and non-homogenous lesions and various dysplastic tissues. All patients received PDT using a 632 nm laser at 500 mW/cm^2^ power density at a dosage of 90–180 J/cm^2^ and with aminolevulinic acid (ALA) used as a photosensitizer. A fixing and restricting complex as well as high laser power density for PDT in oral cavity was applied.

**Results:** An overall response rate of 86.2% was achieved in this study, including 55.2% complete remission and 31.0% partial remission. The only adverse events observed in subjects were transient local ulcer and pain. It is observed the PDT utilizing ALA showed strong effectiveness in patients with moderate to severe dysplasia, as less treatment time per cm^2^ of lesion is required.

**Conclusion:** Topic ALA-PDT is effective to treat oral leukoplakia, especially for that with the presence of dysplasia. A fixing and restricting complex as well as high laser power density for PDT in oral cavity should be considered as an optimal choice.

## Introduction

Oral leukoplakia is a common mucosal pathological lesion of the oral cavity, with high malignant potential (Kramer et al., 1978). A systemic review included more than 1000 patients indicated that its prevalence is between 1.49 and 4.27% (Petti, 2003). Previous studies of leukoplakia cohort showed a wide range of malignant transformation incidence (3–20%) (Bsoul et al., 2005; Pfammatter et al., 2012), which is growing in Chinese population (Shiu et al., 2000; Liu et al., 2010; Lian et al., 2013). Despite various treatment procedures were attempted for the management of oral leukoplakia, there is no clear evidence of effective treatment in preventing malignant transformation and recurrence of oral leukoplakia (Rhodus et al., 2014).

Photodynamic therapy (PDT) is a minimally invasive treatment for local tissue hyperplasia, which was first developed in the 1970s (Lodi et al., 2016). The basic principle of PDT is the simultaneous application of a photosensitizing compound, oxygen and visible light. After the photosensitizing compound penetrates the targeted tissues, a compound-specific, low-power beam of visible light illuminates, and activates the photosensitizing compound (Chau et al., 2017). As a result, the activated compound produces reactive oxygen species (ROS), interrupting crucial cell components and inducing apoptosis and cell death. Therefore, PDT causes minimal damage to surrounding tissues and structures, resulting in good functional and cosmetic outcomes (Konopka and Goslinski, 2007).

The basic principle of PDT is the interaction between a photosensitizer, the appropriate activating wavelength of light, and oxygen. When light illuminates the cells that have taken up an exogenous photosensitizer, the reaction generates ROS that lead to damage of the lesion but minimally affects the surrounding tissue.

Photodynamic therapy has been applied in the treatment of oral leukoplakia for over 10 years, and exhibited encouraging results (Kubler et al., 1998; Vohra et al., 2015). So far, only a few groups recently reported PDT as a treatment option for Chinese oral leukoplakia patients (Ge et al., 2011; Shen et al., 2013), there remain many barriers leading to a significant proportion of oral leukoplakia patients receiving PDT. Here, this study evaluates both its overall clinical efficacy and its adverse effects of PDT with topical application of aminolevulinic acid hydrochloride (ALA) in oral leukoplakia patients, which is meant to provide benchmarks or standards to clinical practice.

## Materials and Methods

### Cell Culture

Cells derived from human tongue squamous cell carcinoma (SCC15 line, ATCC^®^) were grown in Dulbecco’s modified Eagle medium (Sigma Chemical Co., St. Louis, MO, United States)/F12 HAM Nutrient Mixture (Gibco – Invitrogen, Carlsbad, CA, United States) at 1:1 proportion, supplied with 10% fetal bovine serum (Gibco) and 1% antibiotic/antimycotic solution (Gibco). Cells were incubated at 37°C in 90% humidity containing 5% CO_2_ (Yang et al., 2007).

### Temperature Measure

Laser was used to illuminate which based on the different concentration of ALA. The temperature of the growth medium was record, which was obtained by using a fluency of 10 J/cm^2^ (100, 300, and 600 mW, respectively).

### Reactive Oxygen Species (ROS) and Apoptosis Analysis by Cell Counting Kit (CCK)-8 Detection

ROS generation was evaluated with H2DCFDA. human tongue squamous cell carcinoma (SCC15 line, ATCC^®^) were incubated with H2DCFDA (20 μM) in cell culture media at 37°C for 1 h. No internalized dye was washed away with PBS × 3 and fresh media was replaced prior to the PDT. The SCC-15 (ATCC^®^) were exited with 405 nm laser (100 mW) for 3 min and subsequently imaged with confocal microscopy.

Prior to detection, 10 μl CCK-8 liquid was added to each medium and incubated for 1 h. The optical density (OD) was detected at 450 nm using a microplate reader (BioTek ELX808 American) and the average OD was calculated (Liu et al., 2017; Näkki et al., 2017; Rosin et al., 2018).

### Inclusion and Exclusion Criteria

Twenty-nine patients with oral leukoplakia were enrolled from Beijing Stomatological Hospital. Patient eligibility criteria included the following: (1) age between 18 and 80 years old; (2) oral leukoplakia was clinically diagnosed and confirmed using histology (including hyperplasia and dysplasia). Patient exclusion criteria were: (1) pathologically diagnosed with oral carcinoma *in situ* and could tolerate surgery; (2) high risk for local anesthesia; (3) lesions located on the palate or other places not suitable for photosensitizer application; (4) declined to consent to accept PDT therapy; (5) patients with underlying oral cancer. All patients were given oral hygiene instructions and restriction guidelines for smoking, alcohol consumption, and betel nut chewing. Informed consent was obtained from each patient before enrollment. This study was approved by the Institutional Review Board in Peking University School of Stomatology, Stomatological Hospital and Stomatology Research Institute. Application Receipt Number is PKUSSIRB-201416088.

### Clinical Examination

Before PDT treatment, all oral leukoplakia lesions were examined, photographed, and measured. If the lesion was approximately in a regular shape, a periodontal probe was utilized to measure the diameter in both directions. The area of the lesion was calculated by the diameters. Because the treatment was performed one by one, meaning the rectangle with 2 × 2 and the oval with 2 × 2 received the same treatment time. If the lesion was in an irregular shape, it was divided it into several parts. Each part was measured by the method.

### PDT Procedure

ALA was used as the PDT photosensitizer. ALA topical powder and thermosensitive gels were purchased from Fudan-Zhangjiang Bio-Pharmaceutical Co., Ltd., Shanghai, China. 118 mg ALA was dissolved in 0.5 mL gel solution to make 20% ALA gel. PDT was performed using a PDT instrument with a 632 nm He-Ne laser (LH-600 Leiyi Laser Technology Co., Ltd., Tianjin, China) and Silica Fiber (provide by Leiyi Laser Technology Co., Ltd., Tianjin, China).

**FIGURE 1:**
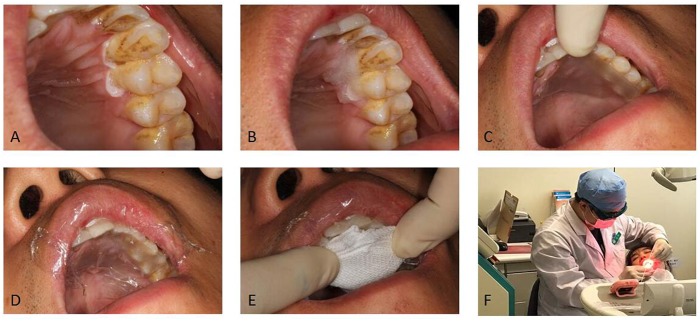
The procedure of administrating topical medications. **(A)** The leukoplakia was dried from gingival mucosa. **(B)** Gel was laid on the lesion. **(C)** A piece of glutinous rice paper was covered. **(D)** Food wrap was covered. **(E)** Gauze was covered. **(F)** Position of patient for medication administration. We have obtained the consent of the patient to publish these photos.

Briefly, 20% ALA gel was directly applied to the oral leukoplakia lesions using cotton pads for 2 h before laser illumination (Figure 1). The patient had written informed consent and agreed to publish the photos. Saliva was removed from the surrounding areas. The medicine was laid on the lesion with cotton wool then covered with a piece of paper (glutinous rice) and food wraps to avoid introduction of saliva to the treatment site. Gauze was used to stabilize the medicine if necessary.

A 395 nm UVA flashlight (Grandoor Electronic Co., Ltd., Shenzhen, China) and a NBP635 narrow band filter (Feiyuda Optoelectronic Technology Co., Ltd., Shenzhen, China) were used to observe the fluorescence reaction ppIX fluorescence at the time of applying the ALA 2 h later (just before laser exposure). We didn’t measure the fluorescence intensity. It was observed mere presence or absence of fluorescence. If the lesion exhibited red fluorescence, the treatment was initiated.

Local oral anesthesia (primacaine) was administered immediately before the laser therapy. Laser therapy was applied at a fluency rate of 600 mW/cm^2^ for 180 s/cm^2^. The rated output power of the equipment was 600 mW/cm^2^. The actual power was measured to be 500 mW/cm^2^. Power was regularly measured to ensure that it was kept constant.

The laser was manually directed at the treatment site, without fiber assisted irradiation or lens-ended device. Light was directed at a spot with a 1 cm diameter. This was repeated at several locations as necessary in order to cover the patient’s whole lesion. Light spot diameter was measured using periodontal probing and visual inspection. Each spot overlapped with adjacent spots by 3–5 mm. Power was regularly measured to ensure that it was kept constant. This equaled to a light exposure 90–180 J/cm^2^ (Kawczyk-Krupka et al., 2012; Rosin et al., 2017), according to the overlapping portion. The duration of each stage of this therapy was 3 min with 1 min intervals between each stage. No treatment session covered a total area exceeding 4 cm^2^ or longer than 3 cm in any direction due to the difficulty of effective moisture exclusion. Neighboring tissues were not exposed to PDT.

After the laser therapy, topical Triamcinolone Acetonide Dental Paste (Bright Future Pharmaceuticals Factory, Hongkong, China) was applied to promote ulcer healing. Local ulcer with pain was reported in 65.5% patients after PDT.

YT308 Digital Thermometer (Jiangsu Yuyue Medical Instruments Co., Ltd., Yancheng, Jiangsu, China) was used to measure the temperature of mucosa during treatment. No apparent change was observed.

### Clinical Evaluation

All patients attended follow-up visits for 3 months after the initial PDT treatment session. The majority of the cases were evaluated by observations, only a few of cases with moderate to severe dysplastic taken biopsy for the second time. The lesion was measured before and after treatment. Lesion response categories were defined as follows (evaluated by observations): complete remission (CR), lack of detectable lesion confirmed by clinical evaluation; partial remission (PR), reduction of lesion size by at least 20% in diameter; no response (NR), reduction of lesion size by less than 20% in diameter. In each follow-up session, the lesions were measured. If lesion size was not reduced by more than 50% after 3 PDT treatments, the patient would not receive further PDT. The patients were taking lycopene.

### Statistical Analysis

Statistical analysis was performed through SPSS 17.2. Data were presented as mean ± SD or number (percentage). Student *t*-test method was used to compare quantitative data in two groups, ANOVA was used to compare quantitative data in multiple groups, and Chi-square test was used to compare nominal (categorical) data between two or more groups. Correlation between demographics and clinical outcome was assessed through Pearson correlation analysis. A *p* value less than 0.05 was considered as statistically significant.

## Results

### *In vitro* Part

There was no significant temperature changed in different ALA concentration exposure to same laser power density. In the same ALA concentration, as the power increased, so does the temperature significant increased (rank-sum test, *P* < 0.05) (Table 1).

**Table 1:** The temperature of different laser power density and ALA concentration ALA-PDT on SCC-15 cell.

Power density (mW)	Temperature of cell culture medium (°C)			

	Blank control	0.1 mg/ml ALA	0.2 mg/ml ALA	0.5 mg/ml ALA
0	28.51 ± 1.12	27.43 ± 2.14	28.01 ± 0.54	26.43 ± 2.56
100	30.12 ± 1.73	31.57 ± 0.38	31.22 ± 2.10	30.99 ± 1.49
300	36.23 ± 1.98	35.33 ± 1.88	37.55 ± 2.41	35.88 ± 1.23
600	43.85 ± 2.06	45.12 ± 0.76	43.23 ± 1.97	44.60 ± 1.44


*Blank control had no ALA.*

The OD of ROS was no significant different between group 1 and 2. Also there were no significant different between group 3, 4, and 5. But the group 3, 4, and 5 were significant lower than the group 1 and 2 (rank-sum test, *P* < 0.05). The same results were seen in the CCK-8 detection (Table 2).

**Table 2:** The results of ROS and CCK-8 in different laser power density ALA-PDT on SCC15 cells.

Group 1		Group 2		Group 3		Group 4		Group 5	
				
ROS	CCK-8	ROS	CCK-8	ROS	CCK-8	ROS	CCK-8	ROS	CCK-8
0.575	0.62	0.592	0.698	0.518	0.512	0.556	0.497	0.631	0.437
0.606	0.639	0.651	0.648	0.42	0.597	0.518	0.452	0.44	0.397
0.655	0.676	0.618	0.566	0.431	0.413	0.486	0.413	0.464	0.38


*Group 1, SCC15 cells without ALA and illuminate; Group 2, SCC15 cells contained 0.5 mg/ml ALA and without illuminate; Group 3, SCC15 cells contained 0.5 mg/ml ALA explored to 100 mW laser power density achieving 10 J/cm^2^; Group 4, SCC15 cells contained 0.5 mg/ml ALA explored to 300 mW laser power density achieving 10 J/cm^2^; Group 5, SCC15 cells contained 0.5 mg/ml ALA explored to 600 mW laser power density achieving 10 J/cm^2^.*

### Demographic Information

A total of 29 oral leukoplakia patients (11 male and 18 female, mean age 56.07 ± 11.01 years) were enrolled in the study. These patients had been diagnosed with oral leukoplakia for 34.69 ± 46.48 months (range: 1–240 months). Of the 29 lesions, 69.0% (20/29) were homogenous and 31.0% (9/29) were non-homogenous. Nine lesions were observed on the cheek, 2 on the gum, 3 on the dorsal surface of the tongue, and 15 on the ventral surface of the tongue. The average lesion diameter was 2.30 ± 1.04 cm (0.8–5.0 cm). 65.5% of the patients (19/29) did not have clinical symptoms or displayed only rough lesion texture as a symptom. 34.5% (10/29) had oral pain or ulcer. Biopsy was performed for all lesions and hematoxylin and eosin (H&E) staining was performed to classify dysplastic pathology. Of the 29 lesions, 34.5% (10/29) were non-dysplastic, 41.4% (12/29) were mildly dysplastic, and 24.1% (7/29) were moderately to severely dysplastic. Detailed demographics of PDT treated patients is summarized in Table 3.

**Table 3:** Demographics of PDT treated patients.

Variable	n (%) or mean ±*SD*
**Overall (*n* = 29)**	
Gender	
Male	11 (37.9%)
Female	18 (62.1%)
Age (year)	56.07 ± 11.01
Oral leukoplakia history (month)	34.69 ± 46.48
Lesions location	
Cheek	9 (31.0%)
Gum	2 (6.9%)
Tongue, dorsal surface	3 (10.3%)
Tongue, ventral surface	15 (51.7%)
Smoke history	
Yes	5 (17.2%)
No	24 (82.8%)
Lesion classification	
Non-homogenous	9 (31.0%)
Homogenous	20 (69.0%)
Clinical symptoms (side effect)	
None or rough lesion texture	19 (65.5%)
Oral pain or local ulcer	10 (34.5%)
Dysplastic pathology	
Non-dysplastic	10 (34.5%)
Mildly dysplastic	12 (41.4%)
Moderately to severely dysplastic	7 (24.1%)
Response to PDT	
Complete remission (CR)	16 (55.2%)
Partial remission (PR)	9 (31.0%)
No remission (NR)	4 (13.8%)
Total lesion area (cm^2^)	3.19 ± 3.16
Average lesion diameter (cm)	2.30 ± 1.04
**Follow-ups (*n* = 25, only CR and NR patients)**	
Number of treatment sessions	4.08 ± 1.73
Total treatment duration (min)	39.92 ± 28.12
Treatment unit duration (min/cm^2^)	19.93 ± 13.85
Recurrence rate in 3 months	3 (12.0%)

### Clinical Outcomes of PDT

**FIGURE 2:**
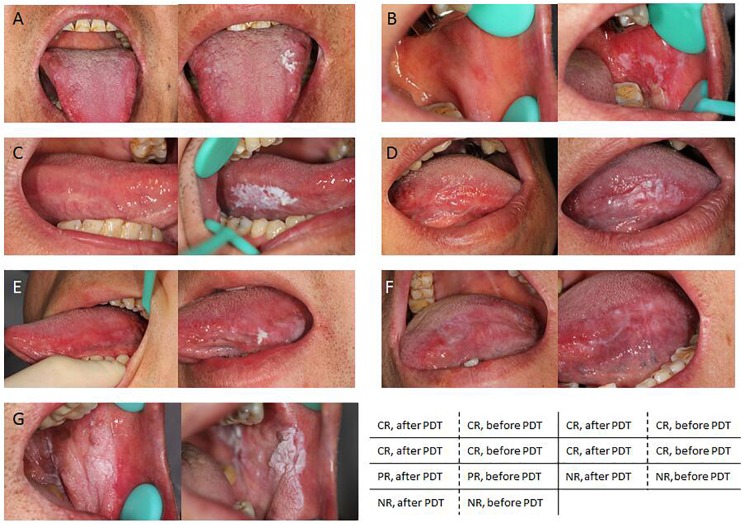
Representative images showing different clinical outcomes. **(A–D)** PDT significantly facilitate smoother skin and an overall improved appearance in patients with complete remission (CR). **(E)** Partial improved appearance in patients with partial remission (PR). **(F,G)** No response was noticed in patients with no remission (NR).

Photodynamic therapy treatment for oral leukoplakia achieved an overall positive response rate of 86.2% (25/29), while the rest patients 13.8% (4/29) did not exhibit any change after treatment (Table 3). Among the 25 responding patients, 64.0% (16/25) of them experienced CR (Figures 2A–D) while the rest of them experienced PR (Figure 2E). Notably, PDT response rate showed significant difference between the homogeneous and non-homogeneous leukoplakia groups (*p* = 0.019, Table 4), suggesting better clinical benefit occurs in homogenous leukoplakia than non-homogeneous leukoplakia (Table 4). In contrast to that, PDT treatment efficacy is comparable between patients with different gender, smoke history or dysplastic pathology (all *p* > 0.05, Table 4). We did observed treatment time is longer for the non-dysplastic leukoplakia than the dysplastic lesions can get remission after PDT, which need more treatment time than the dysplastic lesions (*p* < 0.05, Table 4).

**Table 4:** Comparison of clinical outcomes of patients in different groups.

	Response to PDT			
	
	Complete	Partial	No
Patient grouping	remission	remission	remission	*p* value
Gender				0.330
Male	8 (72.79%)	2 (18.2%)	1 (9.1%)	
Female	8 (44.4%)	7 (38.9%)	3 (16.7%)	
Smoke history				0.749
Yes	2 (40.0%)	2 (40.0%)	1 (20.0%)	
No	14 (58.3%)	7 (29.2%)	3 (12.5%)	
Lesion classification				0.019^∗^
Non-homogenous	2 (22.2%)	6 (66.7%)	1 (11.1%)	
Homogenous	14 (70.0%)	3 (15.0%)	3 (15.0%)	
Clinical symptoms				0.277
None or rough lesion texture	10 (52.6%)	5 (26.3%)	4 (21.1%)	
Oral pain or ulcer	6 (60.0%)	4 (40.0%)	0 (0%)	
Dysplastic pathology				0.359
Non-dysplastic	7 (70.0%)	2 (20.0%)	1 (10.0%)	
Mildly dysplastic	4 (33.3%)	6 (50.0%)	2 (16.7%)	
Moderately to severely dysplastic	5 (71.4%)	1 (14.3%)	1 (14.3%)	

	**Treatment**		
	**unit duration**	**Group**		
	**(min/cm^2^)**	**compression**		***p* value**

Dysplastic pathology group				
I. Non-dysplastic	28.0 ± 14.6	group I vs. II		0.080
II. Mildly dysplastic	17.3 ± 12.2	group I vs. III		0.011^∗^
III. Moderately to severely dysplastic	12.2 ± 8.0	group II vs. III		0.275

*Data is given as n with percentage in the row. ^∗^p < 0.05 (Chi-square test or t-test).*

In total 96 follow-ups were conducted with 25 patients every 2 or 3 weeks. The patients with CR or PR received averagely 4.08 ± 1.73 treatment sessions (Table 3). The unit treatment time per cm^2^ of lesion of these patients was 19.9 ± 13.8 min. During the 3-month follow-up, the recurrence rate was 12% (3/25). All recurrence patients exhibited PR after the initial treatment. Patients with NR showed no signs of deterioration.

### Treatment Duration Is Associated With Dysplastic Pathology

In addition, necessary treatment duration was found to be related to the dysplasia severity inside the lesions. The unit durations for non-dysplastic lesions and mild dysplastic lesions were 28.0 ± 14.6 min/cm^2^ and 17.3 ± 12.2 min/cm^2^, respectively. The unit treatment duration for moderate to severe dysplastic lesions was 12.2 ± 8.0 min/cm^2^ (Table 4), which is significantly shorter than non-dysplastic dysplastic lesions (*p* < 0.05, Student’s *t*-test). No statistical difference was observed between mild dysplastic lesions with moderate to severe or non-dysplastic lesions (Table 4). Further analysis showed the total treatment duration was not associated with age, gender, disease history, and lesion classification (data not shown).

**FIGURE 3:**
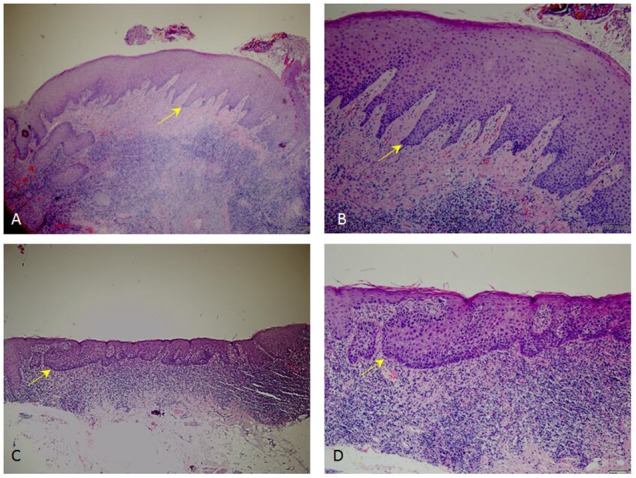
Representative H&E staining images showing dysplastic pathology. **(C,D)** Pathological sections of moderate to severe dysplastic lesions from patient before PDT. **(A,B)** Pathological sections of non-dysplastic tissues from patient received PDT. **(A,C)**, 4× magnification; **(B,D)**, 10× magnification.

At the baseline, dysplastic tissues were most likely to be located in non-keratinized area, including cheeks and the ventral side of the tongue, rather than gums and the dorsal side of the tongue, which are keratinized area (*p* < 0.05, Table 5). As an example, our H&E staining result showed successful PDT treatment can substantially reduce dysplastic region inside of the lesions that positioned on ventral side of the tongue (Figure 3).

**Table 5:** Comparison of lesion location in patient with different dysplastic pathology.

	Lesion location		
	
Patient grouping	Keratinized area	Non-keratinized area	*p* value
Dysplastic pathology			0.016^∗^
Non-dysplastic	4 (40.0%)	6 (60.0%)	
Mildly dysplastic	1 (8.3%)	11 (91.7%)	
Moderately to severely dysplastic	0 (0%)	7 (100%)	


*Data is given as n with percentage in the row. ^∗^p ( <0.05 (Chi-Square).*

### Adverse Events and Side Effects

Local ulcers and oral pain were observed in 65.6% patients (19/29), which were typically resolved within 2 weeks (Table 3). In contrast to that, 10.3% (3/29) patients who were all over 60 years of age exhibited ulcer and pain symptoms beyond a 2-week period. Their ulcer symptom lasted more than 2 weeks after PDT treatment. Pain or bleeding from the ulcers impaired drug application. Therefore, for these patients, the subsequent treatment interval was changed from an initial schedule of every 2 weeks to every 3 weeks.

The clinical side effects of ulcer and pain were examined and showed association with oral leukoplakia history, as well as lesion classification. Patients with a long history of oral leukoplakia, and those who had non-homogenous lesions, were more likely to develop ulcer and pain (all *p* < 0.05, Table 6).

**Table 6:** Comparison of side effect in different patient groups.

	Adverse events and side effects		
	
	None or rough	Oral pain or
Patient grouping	lesion texture	local ulcer	*p* value
Oral leukoplakia history (month)	18.68 ± 13.93	65.01 ± 69.03	0.003^∗^
Total treatment duration (min)	31.80 ± 16.45	52.10 ± 37.60	<0.001^∗^
Total lesion area (cm^2^)	3.12 ± 3.78	3.29 ± 2.08	0.334
Lesion classification			0.022^∗^
Non-homogenous	16 (80.0%)	4 (20.0%)	
Homogenous	3 (33.3%)	6 (66.7%)	
Dysplastic pathology			0.855
Non-dysplastic	7 (70.0%)	3 (30.0%)	
Mildly dysplastic	8 (66.7%)	4 (33.3%)	
Moderately to severely dysplastic	4 (57.1%)	3 (42.9%)	


*Data is given as n with percentage in the row. ^∗^p < 0.05 (ANOVA or Chi-Square).*

## Discussion

This retrospective study evaluated the therapeutic efficacy and the clinical performance of topical ALA-PDT in Chinese patients with oral leukoplakia. First, the PDT demonstrated significant efficacy with high positive response rate. Second, a new method of topical drug application, a fixing and restricting complex that can isolate moisture was used for the ALA-PDT treatment in oral cavity. Third, treatment session and interval between sessions was planned carefully. Fourth, a high laser power density was used. Fifth, the relationship between therapeutic outcome and the clinical types, mucosal keratinization state, and dysplastic grade of the lesions was investigated separately.

### Effectiveness of PDT

In a previous double-blind controlled trial performed with 46 Japanese oral leukoplakia patients, Beta-carotene and vitamin C were not effective for clinical remission (Nagao et al., 2015). This study demonstrated a high response rate of 86.2%, including 55.2% CR and 31.0% PR. Overall, these results indicated successful and effective outcomes. This rate is agreed with reports from non-Asian cohorts with a successful rates ranges from 77.3 to 83.3% (Sieron et al., 2003; Jerjes et al., 2011; Shafirstein et al., 2011; Pietruska et al., 2014). Importantly, the response rate of PDT we observed is higher than the response rate (<40%) reported by another study investigated 237 lesions from Japanese patients selected for non-surgical therapy (Kuribayashi et al., 2015).

### High Laser Power Density

The laser dose and the PDT laser power density used in this study was inherited from published report (Sieron et al., 2003), although this setting of laser power is relatively higher than many other research (Sieron et al., 2001; Yu et al., 2008, 2009; Chang and Yu, 2014). The high dose was only be used in obstructing esophageal cancer (Sieron et al., 2003). We have surmised that one major reason for the prevalence of lower-powered laser use in the reported treatments is that higher powered lasers applied to PDT may induce significantly more pain, especially when the power exceeds 300 mW/cm^2^. Clinical methods to mitigate this include intermittent illumination, local anesthesia, and cooling. In this study, local anesthesia was performed using primacaine and limited the treatment area for each session, which improved patient tolerability.

Some studies have also reported that high power PDT may exhaust local oxygen content and affect clinical outcomes, because oxygen is required to generate ROS after laser illumination. From our *in vitro* study, that showed there were no significant differences of ROS and apoptosis between power density groups. Meanwhile, the PDT intensity was optimized and the he similar clinical efficacy was observed. This is partially due to blood vessels are more abundant in oral mucosa, the oxygen supply is able to quickly replenish during PDT. Higher power PDT can reduce treatment time and cover a broader lesion area. As a result, open mouth time is decreased to improve tolerability. Although 65.6% patients reported ulcer and pain after treatment, most of them recovered within 2 weeks, aside from the previously discussed elderly patients who required 3 weeks to recover (Jerjes et al., 2011; Shafirstein et al., 2011). The data from study indicated that patients over 60 years old who require treatment in an area larger than 1 cm in diameter, treatment should be performed every 3 weeks. The suitable interval may be less than 2 weeks for patients who do not show significant side effects.

**FIGURE 4:**
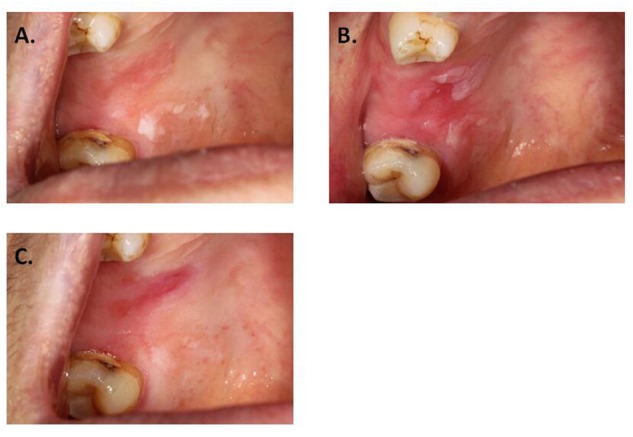
Local ulceration after PDT. **(A)** Leukoplakia on hard palate, before applying ALA. **(B)** Before laser exposure. **(C)** One week after PDT.

Some previous studies mentioned the post-operation pain (Yu et al., 2008; Jerjes et al., 2011). Although few studies mentioned the ulcer after ALA-PDT, we could see the ulceration from the pictures in the articles (Lin et al., 2010; Chang and Yu, 2014). However, the different laser dense in this study and in the literatures, there was a postoperative reaction of pain and ulcer. Furthermore, this case in the study confirmed that the high-powered laser light wouldn’t lead ulcer. In this case, PDT treatment applied on the right hard palate but only part of the lesion appeared ulcer (Figure 4).

### The Influence of Therapeutic Outcomes

In this study, four patients did not respond to PDT, including one patients with non-dysplastic lesion, two with mild dysplastic lesion, and one with moderate dysplastic lesion. In the non-dysplastic patient, the lesion was located in the keratinized area. The most severe area was treated. The lesion area was decreased following PDT treatment and mildly decolorized, however, it did not reach the remission standard (Figure 2G). It is possible that the photosensitizing compound did not penetrate in the keratinized area, affecting its expected clinical outcomes (Fan et al., 1996). Neither patient experienced significant side effects (ulcer or pain) after the treatment, suggesting that the lesions did not receive excessive photosensitization. For the remaining two patients that did not respond to treatment, the classic lesions were resected during biopsy, and the remaining lesions lacked a clear boundary (Figure 2F). The remaining lesions had week fluorescence reaction, partially due to the less dysplastic, or badly fixing and restricting experience, therefore had poor effect to PDT. Though we have included a decent sample size in this study, future validation studies will be needed to ensure that these result are sufficiently represented to enable more rigorous statistical results and conclusions. Multicenter cooperation with larger sample enrollment for multiple comparisons will be included in future study.

## Conclusion

In summary, we found that topic ALA-PDT is effective to treat oral leukoplakia, with better outcomes in the presence of dysplasia. A fixing and restricting complex for PDT in oral cavity is considered an optimal choice. By using the high laser power density on oral mucosal lesion, a high response rate can be achieved with the short treatment time and better tolerability for the patients. For leukoplakia of non-homogeneous type or in keratinized area, the optimized procedure conditions will need to be explored further. The long-term outcomes of this treatment of leukoplakia in muilti-center study will be the future focus.

## Ethics Statement

This study was carried out in accordance with the recommendations of The Application of Photodynamic Therapy in Oral Leukoplakia, IRB of Peking University School and Hospital of Stomatology. The protocol was approved by the IRB of Peking University School and Hospital of Stomatology. All subjects gave written informed consent in accordance with the Declaration of Helsinki.

## Author Contributions

YH did the majority of the clinical treatment and wrote the article. HL designed the research and modified the article. SX, JJ, and XL did the some of the clinical treatment and collated the data. HH and XiaW gave some advise. XinW did the *in vitro* test part of this article and drafting part of the article.

## Conflict of Interest Statement

The authors declare that the research was conducted in the absence of any commercial or financial relationships that could be construed as a potential conflict of interest.

## References

[B1] Bsoul S. A. Huber M. A. Terezhalmy G. T. (2005). Squamous cell carcinoma of the oral tissues: a comprehensive review for oral healthcare providers. *J. Contemp. Dent. Pract.* 6 1–16. 16299602

[B2] Chang Y. C. Yu C. H. (2014). Successful treatment of oral verrucous hyperplasia with photodynamic therapy combined with cryotherapy–report of 3 cases. *Photodiagnosis Photodyn. Ther.* 11 127–129. 10.1016/j.pdpdt.2014.02.003 24561304

[B3] Chau L. Jabara J. T. Lai W. Svider P. F. Warner B. M. Lin H. S. (2017). Topical agents for oral cancer chemoprevention: a systematic review of the literature. *Oral Oncol.* 67 153–159. 10.1016/j.oraloncology.2017.02.014 28351570

[B4] Fan K. F. Hopper C. Speight P. M. Buonaccorsi G. MacRobert A. J. Bown S. G. (1996). Photodynamic therapy using 5-aminolevulinic acid for premalignant and malignant lesions of the oral cavity. *Cancer* 78 1374–1383. 10.1002/(SICI)1097-0142(19961001)78:7<1374::AID-CNCR2>3.0.CO;2-L 8839541

[B5] Ge L. Wu Y. Wu L. Y. Zhang L. Xie B. Zeng X. (2011). Case report of rapidly progressive proliferative verrucous leukoplakia and a proposal for aetiology in mainland China. *World J. Surg. Oncol.* 9:26. 10.1186/1477-7819-9-26 21352571 PMC3056810

[B6] Jerjes W. Upile T. Hamdoon Z. Mosse C. A. Akram S. (2011). Hopper C. Photodynamic therapy outcome for oral dysplasia. *Lasers Surg. Med.* 43 192–199. 10.1002/lsm.21036 21412802

[B7] Kawczyk-Krupka A. Waśkowska J. Raczkowska-Siostrzonek A. Kościarz-Grzesiok A. Kwiatek S. Straszak D. (2012). Comparison of cryotherapy and photodynamic therapy in treatment of oral leukoplakia. *Photodiagnosis Photodyn. Ther.* 9 148–155. 10.1016/j.pdpdt.2011.12.007 22594985

[B8] Konopka K. Goslinski T. (2007). Photodynamic therapy in dentistry. *J. Dent. Res.* 86 694–707. 10.1177/154405910708600803 17652195

[B9] Kramer I. R. Lucas R. B. Pindborg J. J. Sobin L. H. (1978). Definition of leukoplakia and related lesions: an aid to studies on oral precancer. *Oral Surg. Oral Med. Oral Pathol.* 46 518–539. 10.1016/0030-4220(78)90383-3 280847

[B10] Kubler A. Haase T. Rheinwald M. Barth T. Mühling J. (1998). Treatment of oral leukoplakia by topical application of 5-aminolevulinic acid. *Int. J. Oral Maxillofac. Surg.* 27 466–469. 10.1016/S0901-5027(98)80040-4 9869290

[B11] Kuribayashi Y. Tsushima F. Morita K. I. Matsumoto K. Sakurai J. Uesugi A. (2015). Long-term outcome of non-surgical treatment in patients with oral leukoplakia. *Oral Oncol.* 51 1020–1025. 10.1016/j.oraloncology.2015.09.004 26410021

[B12] Lian Ie B. Tseng Y. T. Su C. C. Tsai K. Y. (2013). Progression of precancerous lesions to oral cancer: results based on the Taiwan National Health Insurance Database. *Oral Oncol.* 49 427–430. 10.1016/j.oraloncology.2012.12.004 23273345

[B13] Lin H. P. Chen H. M. Yu C. H. Yang H. Wang Y. P. Chiang C. P. (2010). Topical photodynamic therapy is very effective for oral verrucous hyperplasia and oral erythroleukoplakia. *J. Oral Pathol. Med.* 39 624–630. 10.1111/j.1600-0714.2010.00935.x 21054548

[B14] Liu L. Zhao W. M. Yang X. H. Sun Z. Q. Jin H. Z. Lei C. (2017). Effect of inhibiting Beclin-1 expression on autophagy, proliferation and apoptosis in colorectal cancer. *Oncol. Lett.* 14 4319–4324. 10.3892/ol.2017.6687 28989537 PMC5620484

[B15] Liu W. Wang Y. F. Zhou H. W. Shi P. Zhou Z. T. Tang G. Y. (2010). Malignant transformation of oral leukoplakia: a retrospective cohort study of 218 Chinese patients. *BMC Cancer.* 10:685. 10.1186/1471-2407-10-685 21159209 PMC3009685

[B16] Lodi G. Franchini R. Warnakulasuriya S. Varoni E. M. Sardella A. Kerr A. R. (2016). Interventions for treating oral leukoplakia to prevent oral cancer. *Cochrane Database. Syst. Rev.* 7:CD001829. 10.1002/14651858.CD001829.pub4 27471845 PMC6457856

[B17] Nagao T. Warnakulasuriya S. Nakamura T. Kato S. Yamamoto K. Fukano H. (2015). Treatment of oral leukoplakia with a low-dose of beta-carotene and vitamin C supplements: a randomized controlled trial. *Int. J. Cancer* 136 1708–1717. 10.1002/ijc.29156 25156040

[B18] Näkki S. Martinez J. O. Evangelopoulos M. Xu W. Lehto V. P. Tasciotti E. (2017). Chlorin e6 functionalized theranostic multistage nanovectors transported by stem cells for effective photodynamic therapy. *ACS Appl. Mater. Interfaces* 19 23441–23449. 10.1021/acsami.7b05766 28640590 PMC5565768

[B19] Petti S. (2003). Pooled estimate of world leukoplakia prevalence: a systematic review. *Oral Oncol.* 39 770–780. 10.1016/S1368-8375(03)00102-7 13679200

[B20] Pfammatter C. Lindenmüller I. H. Lugli A. Filippi A. Kühl S. (2012). Metastases and primary tumors around dental implants: a literature review and case report of peri-implant pulmonary metastasis. *Quintessence Int.* 43 563–570. 22670251

[B21] Pietruska M. Sobaniec S. Bernaczyk P. Cholewa M. Pietruski J. K. Dolińska E. (2014). Clinical evaluation of photodynamic therapy efficacy in the treatment of oral leukoplakia. *Photodiagnosis Photodyn. Ther.* 11 34–40. 10.1016/j.pdpdt.2013.10.003 24211597

[B22] Rhodus N. L. Kerr A. R. Patel K. (2014). Oral cancer: leukoplakia, premalignancy, and squamous cell carcinoma. *Dent. Clin. North Am.* 58 315–340. 10.1016/j.cden.2013.12.004 24655525

[B23] Rosin F. C. Barcessat A. R. Borges G. G. Ferreira L. G. Corrêa L. (2017). Vascular alterations after photodynamic therapy mediated by 5-aminolevulinic acid in oral leukoplakia. *Lasers Med. Sci.* 32 379–387. 10.1007/s10103-016-2127-0 28004205

[B24] Rosin F. C. P. Teixeira M. G. Pelissari C. Corrêa L. (2018). Resistance of oral cancer cells to 5-ALA-mediated photodynamic therapy. *J. Cell Biochem.* 119 3554–3562. 10.1002/jcb.26541 29227548

[B25] Shafirstein G. Friedman A. Siegel E. Moreno M. Bäumler W. Fan C. Y. (2011). Using 5-aminolevulinic acid and pulsed dye laser for photodynamic treatment of oral leukoplakia. *Arch. Otolaryngol. Head Neck Surg.* 137 1117–1123. 10.1001/archoto.2011.178 22106236

[B26] Shen L. Qing X. Pingping L. Guoyz Z. (2013). Efficacy of krypton laser photodynamic therapy for oral mucosa dysplasia in 9,10-dimethyl-1,2-benzanthracene-treated hamsters. *Oncol. Lett.* 6 1358–1362. 10.3892/ol.2013.1554 24179524 PMC3813801

[B27] Shiu M. N. Chen T. H. H. Chang S. H. Hahn J. (2000). Risk factors for leukoplakia and malignant transformation to oral carcinoma: a leukoplakia cohort in Taiwan. *Br. J. Cancer* 82 1871–1874. 10.1054/bjoc.2000.1208 10839305 PMC2363234

[B28] Sieron A. Adamek M. Kawczyk-Krupka A. Mazur S. Ilewic L. (2003). Photodynamic therapy (PDT) using topically applied delta-aminolevulinic acid (ALA) for the treatment of oral leukoplakia. *J. Oral Pathol. Med.* 32 330–336. 10.1034/j.1600-0714.2003.00068.x 12787039

[B29] Sieron A. Namyslowski G. Misiolek M. Adamek M. Kawczyk-Krupka A. (2001). Photodynamic therapy of premalignant lesions and local recurrence of laryngeal and hypopharyngeal cancers. *Eur. Arch. Otorhinolaryngol.* 258 349–352. 10.1007/s004050100347 11699825

[B30] Vohra F. Al-Kheraif A. A. Qadri T. Hassan M. I. Ahmed A. Warnakulasuriya S. (2015). Efficacy of photodynamic therapy in the management of oral premalignant lesions. A systematic review. *Photodiagnosis Photodyn. Ther.* 12 150–159. 10.1016/j.pdpdt.2014.10.001 25315968

[B31] Yang T. H. Chen C. T. Wang C. P. Lou P. J. (2007). Photodynamic therapy suppresses the migration and invasion of head and neck cancer cells in vitro. *Oral Oncol.* 43 358–365. 10.1016/j.oraloncology.2006.04.007 16920382

[B32] Yu C. H. Chen H. M. Hung H. Y. Cheng S. J. Tsai T. Chiang C. P. (2008). Photodynamic therapy outcome for oral verrucous hyperplasia depends on the clinical appearance, size, color, epithelial dysplasia, and surface keratin thickness of the lesion. *Oral Oncol.* 44 595–600. 10.1016/j.oraloncology.2007.08.016 18203648

[B33] Yu C. H. Lin H. P. Chen H. M. Yang H. Wang Y. P. Chiang C. P. (2009). Comparison of clinical outcomes of oral erythroleukoplakia treated with photodynamic therapy using either light-emitting diode or laser light. *Lasers Surg. Med.* 41 628–633. 10.1002/lsm.20841 19816916

